# Chromatin remodeling induced by histone deacetylase inhibitors (HDACis) in HeLa, NIH 3T3 and HepG2 cells

**DOI:** 10.1186/1756-8935-6-S1-P17

**Published:** 2013-03-18

**Authors:** Marina B Felisbino, Maria SV Gatti, Giovana MB Veronezi, Wirla MSC Tamashiro, Maria LS Mello

**Affiliations:** 1 Dept. of Structural and Functionai Biology, Unicamp, Campinas, 13083-862 Brazil; 2 Dept. of Genetics, Evolution and Bioagents, Unicamp, Campinas, 13083-862 Brazil

## Background

In eukaryotic cells, chromatin results from the association of DNA with nuclear proteins packaged into hierarchies of organization. The DNA packaging into chromatin is generally recognized as a major mechanism by which the access of genomic DNA is restricted. The physical barrier to the underlying DNA is precisely regulated, at least in part, by the post-translational modifications of histones. Two of the most studied modifications are the acetylation and deacetylation of lysine residues in the nucleosome core histones, under the control of histone acetyltransferases and histone deacetylases, respectively. HDACis such as valproic acid (VPA) and trichostatin A (TSA) induce acetylation of histones and non-histone proteins, which is correlated with nucleosome remodeling and transcriptional activation [1]. In some cases the DNA methylation state and presence of chromatin-associated non-histone proteins are also affected by HDACis [2,3]. Here we investigated by image analysis procedures whether VPA and TSA, in consequence of their epigenetic action, would affect chromatin supraorganization in different cell lines.

## Materials and methods

HeLa and HepG2 tumoral cells and non-tumoral NIH 3T3 fibroblasts were cultivated for 24 h, treated with 0.05, 0.5 and 1.0 mM VPA or 10, 20 and 100 ng/mL TSA (1-24 h), and subjected to the Feulgen reaction. The textural changes in the chromatin structure of these cells were studied by scanning microspectrophotometry [4]. Decrease in HDAC activity and increase in acetylation of histones H3 and H4 were also considered. The effect of the HDACis on DNA methylation of HeLa cells was investigated by image analysis. HDACi-induced depletion of the heterochromatin protein 1 (HP1) from NIH 3T3 cells was investigated by immunocytochemistry.

## Results

Nuclear areas covered by “condensed” chromatin, which were abundant in untreated controls, appeared reduced in VPA- and TSA-treated cells. Scatter diagrams in which nuclear relative areas covered with condensed chromatin (Sc %) matched the level of textural contrast between condensed and non-condensed chromatin (AAR), revealed decreased Sc% values with increasing AAR values under all the tested treatments. Work in progress suggests DNA demethylation in HeLa cells. Heterochromatin unpackaging and depletion of HP1 from heterochromatin were detected in most NIH 3T3 cell nuclei.

## Conclusions

Changes in chromatin supraorganization are induced in the cell types analyzed after inhibition of histone deacetylases by VPA and TSA, possibly accompanied by changes in gene expression. The chromatin remodeling promoted by these HDACis is also suggested to be affected by DNA demethylation in HeLa cells and histone H3K9me3 hypomethylation in NIH 3T3 cells.
